# Using the coaches voice to improve the representation and experience of females in coaching: a Gaelic games perspective

**DOI:** 10.3389/fspor.2024.1436226

**Published:** 2024-09-11

**Authors:** T. Haughey, A. Graffin, P. Donnelly, B. McGrane, P. McGourty, N. Stapleton, A. Moore, N. Williams, W. Harmon, P. Horgan, A. Lane

**Affiliations:** ^1^ Sport and Exercise Research Institute, Ulster University, Belfast, Ireland; ^2^ Sports & Societies, Dundalk IT etc, Dundalk Institute of Technology, Dundalk, Ireland; ^3^ Department of Health and Nutritional Sciences, Atlantic Technological University, Sligo, Ireland; ^4^ Strategic National Governing Body Programmes & Women in Sport Lead, Sport Ireland, Dublin, Ireland; ^5^ Sport Northern Ireland, Belfast, Ireland; ^6^ Coach Development, The Camogie Association, Dublin, Ireland; ^7^ Coach Development, Ladies Gaelic Football Association, Dublin, Ireland; ^8^ Department of Coaching and Games Development, Gaelic Athletic Association, Dublin, Ireland; ^9^ SHE Research, Department of Sport and Health Sciences, Technological University of the Shannon, Athlone, Ireland

**Keywords:** Gaelic games, female coaches, culture, education, development

## Abstract

**Background:**

Female coaches across all sports and levels are underrepresented on a global scale, existing as peripheral figures on the coaching landscape. This is evident in an Irish context, with a recent report suggesting that just 18.7% of coaches in Gaelic games are female. The reasons for lower levels of female involvement in coaching have been widely documented, and include females feeling undervalued or under-appreciated, lacking confidence, and experiencing a lack of respect, gender stigmatism, and unconscious bias, within unsupportive organisational cultures. The purpose of this research was to examine the impact of structural and cultural factors on female coaches’ lived experiences of coaching in Gaelic games.

**Methods:**

Following ethical approval, 8 online semi-structured focus groups with 38 female coaches from 5 cohorts; generic, inactive, fulltime paid, coach developers, and cross code coaches were conducted. The data were analysed using thematic analysis. Following transcription, codes and quotes relevant to the main research questions in the study were collated and assessed with reference to the Ecological Intersectional Model.

**Discussion:**

Through an iterative process of analysis and interpretation, four key themes, and 13 sub themes were constructed, shaped, and reshaped by the research team. These reflected personal factors, coaching contexts, organisational supports and societal influences that impacted on the lived experiences of female coaches in Gaelic games. These included many barriers and challenges experienced personally and within the organisational culture of Gaelic games that inhibit female involvement and full engagement across the coaching pathway. Leaders within Gaelic games should consider mentorship and networking; development of holistic coaching environments; and greater flexibility in coach education to increase and retain representation of females in coaching.

## 1 Introduction

### 1.1 Context for females in coaching

The 2024 Paris Olympics are set to achieve gender-equal participation for the first time, marking a significant milestone in the promotion of female engagement in sports (1). Additionally, recent studies indicate a positive trend in female participation in youth sport, although these numbers remain lower than those of their male counterparts (2, 3). Despite these gains in participation, there is a noticeable gap in the progression of women into roles such as officiating, leadership, and particularly coaching (4–7), which is concerning given that achieving gender equality in sport requires improvement in sport as a whole (8). In any case, it remains that female coaches are a minority in most sports (9, 10) and females remain peripheral figures at all levels in coaching, particularly in high performance sport, where masculine approaches to coaching, and recruitment of male coaches often prevail (3, 11). At the same time, a more player centred approach to coaching, which is likely to be endorsed to a greater extent by female coaches, is favoured amongst coaches, athletes, parents and sporting organisations alike (11), and is likely to positively impact retention, particularly in youth sport (12).

Trust and providing more support for female coaches is an important strategy to breaking down traditional, old fashioned approaches to coaching, and eliciting more favourable wellbeing and performance outcomes in coaches and athletes (13, 14). Indeed, it is important that sporting organisations adopt systems to support the continuous development of all sport coaches, to ensure they accrue the relevant knowledge, skills and competencies to best fulfil their role (15, 16). In turn, Lara-Bercial et al., (17) emphasised the need to develop coaching as a profession, and to consider factors such as recruitment, education, development, support and recognition to ultimately develop an overall coaching system that will prioritise inclusion, quality and safety for everyone involved in the coaching process. However, gaps in knowledge regarding the specific needs of females involved in coaching, in specific contexts, are limiting the ability to deliver change for female coaches within the overall sport system (18, 19). Research must also “move away from barrier work and focus on what factors support women coaches and help them not only survive but thrive” [(20); p. 137].

### 1.2 Theoretical approaches underpinning research on females in coaching

Ajzen's “Theory of Planned Behaviour” (TPB) is a concept utilised to help understand and predict human intentions and behaviours which are important in supporting female engagement in coaching. Ajzen (21) discussed how attitudes, subjective norms, and perceived behavioural control impact behaviour. “Attitudes towards a behaviour” is the extent to which action of the behaviour is positively or negatively valued by an individual (21, 22). “Subjective norms” refer to the social norms and perceived pressures from society to engage in a certain behaviour or not and “perceived behavioural control” is the perception of a person's own ability to perform and execute a behaviour (22). In many sport settings, subjective norms are influential in coaching contexts, given the traditional position of sport as a male preserve. Changing such cultural norms are met with great resistance and challenge because it requires changing the fundamental values that conflict with the existing ways of “doing things” (23). Sagas et al., (24) utilised the TPB to examine the factors influencing a coach's intention to pursue a head coaching position within a 3-year period. Findings showed that a female coach's intention to pursue their involvement in sport is shaped by a combination of attitudes toward, social referents of, and confidence about pursuing a coaching position. TPB is strongly linked to Banduras’ concept of self-efficacy (25, 26) which when conducting research from a gendered perspective is linked to ideologies in sport that rank male abilities as superior to females, therefore implying that “women's coaching competencies are then unfavourably judged and underappreciated” [(27), pg. 19]. Equally, self-efficacy is a key personal attribute within the coaching profession, as an indicator of self-belief and self-confidence. Williams and Williams [(28), pg. 455] stated that “individuals with high levels of self-efficacy approach difficult tasks as challenges to master rather than as threats to be avoided”, which is important in the context of females working in often challenging coaching environments.

At the same time, coaching is an inherently social process (29), and there are many frameworks that can be referenced to support understanding of the position of female coaches in broader coaching and sporting contexts. Bronfenbrenner's ecological systems theory has been utilised by numerous researchers (9, 30–32) to describe the multidimensional barriers that are present within sport, which impact females in coaching. Subsequent findings reveal lack of self-efficacy and assertiveness, an old boys’ club, bullying/harassment, low pay, homophobia, masculine hegemony, leadership stereotypes, and gender assumptions as challenges for females in coaching. McGinty-Minister et al., (33) adopted the ecological model to make sense of coaches’ experiences in sport, using methods employed by LaVoi and Dutove (9). In this work, the lived experiences of female coaches and subsequent perceptions of this experience varied, thereby showcasing the connections between an individual, their environment, and society in general. It is therefore important to recognise that a female's experience working in sport is complex and can be affected by multiple levels of their environment, individual characteristics as well as societal culture and values. Indeed, other previous research has shown that historically there is a tradition that women are seen as domesticated and maternal (27), consequently contributing to expectations that the typical “hands on” coaching roles should be fulfilled by males and pastoral roles by females (34). It has also been found that many of the challenges experienced by female coaches are reflective of such instances of gender discrimination, unconscious bias, unequal expectations (35) and under appreciation of performance of females in coaching (27, 36).

In summary, when considering a model or theory to support and extend opportunities for female involvement in coaching, an ecological systems approach allows for the consideration of the interrelatedness between systems and how they intertwine to influence and shape experiences (37, 38). Norman et al*.* [(36), p. 395] argued, the underrepresentation of women coaches must be expressed as “a symptom, or an outcome of a deeper issue, rather than the problem in itself.” Despite this, too often, actions or discussion around increasing the representation and improving the experiences of females in coaching, and indeed in other areas of sport, are underpinned by a “fix the women” approach without due consideration of the wider sport system. In turn, it is also important to consider female coaching in specific settings and contexts to develop bespoke actions and targets (11).

### 1.3 Irish and Gaelic games context

In general, it appears that many female coaches in Ireland have faced negative experiences and challenges due to their gender. There are major problem areas which include; (i) dealing with male coaches, male parents, and males in positions of management, (ii) bullying, (iii) not being taken seriously, (iv) lack of respect, (v) being seen as less knowledgeable and (vi) gender stigmatism which provides evidence of a hierarchical power dynamic (39, 40). It is therefore not surprising that recent research conducted by Sport Ireland (41) suggests that men are almost twice as likely to volunteer as a coach than women. Most female coaches are working in either a club or school environment (39) while among sports who employ high performance coaches, less than one third who fulfil these roles are females (40). Braithwaite (42) stated that there appears to be a “glass ceiling” that prevents progression for female coaches which Norman et al. (18) argued is “a concrete ceiling, it's not even glass” due to the organisational sporting cultures that appear to prevent the progression of female coaches. This is none more evident than within the Irish sport of Gaelic games. Gaelic games compromises of six different sports which include; Hurling, Gaelic Football, Camogie, Ladies Gaelic Football, Rounders and Handball. Gaelic games activity takes place in grassroots club settings across the island of Ireland and in representative contexts where players are selected to play on inter-county teams, with talented youth and elite adult players taking part in national competitions. The family of Gaelic games are indigenous to Irish sporting culture and are recognised as the most played sports in Ireland among youth (43). Gaelic games are governed by three separate national governing bodies (NGBs); one for males and two for females. The GAA, who oversee male participation in Gaelic games, is the largest community and sporting organisation in Ireland and since its foundation in 1884 has remained as an amateur association with a volunteer ethos that strives to promote Irish identity (44). The sister organisations of the Camogie Association (CA), founded in 1904, and the Ladies Gaelic Football Association (LGFA), founded in 1974, govern the sports of Camogie and Ladies Gaelic Football and they hold similar core values; to foster a clear sense of community identity, to uphold the amateur status and to promote inclusivity to all.

In recent years there has been a growing impetus to amalgamate the three parties together to operate as one single Gaelic games organisation, underpinned by equality (45). In the interim, the organisations are collaborating particularly in the area of coaching and games, developing a “Gaelic Games Player Pathway” and a “Gaelic Games Coach Pathway”, with a vision to create an effective player development and coaching environment within all Gaelic games’ codes. These positive partnerships and collaborations are promising for future generations, but many gendered elements still remain in Gaelic games. For example, the GAA have an almost ten times higher operating budget than the LGFA and CA (45), more males actively participate in the sports than females (43), and the majority of playing facilities are owned and operated by the GAA, which is problematic in the face of demand from female participants (46). From a coaching perspective, females are under-represented, at 18.7% of the overall coaching workforce (47). Reasons for this are not well understood as to the authors knowledge, just one study, Hogan et al. (48) has addressed female coaching in Gaelic games. The authors used La Voi's (49) Ecological-Intersectional Model (EIM) to explore the experiences and perceptions of volunteer female Gaelic football coaches in Ireland. This framework acknowledges the system of personal relationships and structures within social environments and was used in this instance to understand the influences and complex issues that impacted females in their coaching career. Findings indicated that on an interpersonal level, support from home was an essential component for female coaches to begin or to continue volunteer coaching, with a supportive coaching environment also important to attract and retain female coaches.

Due to the overall paucity of research on female coaches in Gaelic games, this research aims to examine female coaches lived experiences in Gaelic games, and the structural and cultural factors which impact their active involvement in this specific coaching context using an ecological systems model.

The planned objectives of this study were;

a. To identify key determinants of participation and non-participation by female coaches at various levels in Gaelic games.
b. To obtain a better understanding of the challenges and barriers facing female coaches involved at various levels in Gaelic games.
c. To deeper understand the levels of organisational culture within the family of Gaelic games.
d. To consider the implications for policy, strategy, and programme development in order to increase levels of female coach engagement and involvement in Gaelic Games at all levels.

## 2 Materials and methods

### 2.1 Study design

Sports coaching is a practical, social activity that can be characterised as complex, uncertain, singular and with conflicting values (50). Due to this dynamic context and multi-faceted nature of sports coaching, qualitative research methods were adopted. Focus groups were used to facilitate collective discussion of participants beliefs, experiences, attitudes, behaviours, and interactions in coaching (51, 52). From a philosophical perspective an interpretivist, constructivist research paradigm was considered to best suit this study where knowledge gained was co-constructed involving the researched (i.e., the research participants/coaches), and the research team. Additional quantitative data from the 2020 “Coaching and Coach Education in Gaelic Games: A Baseline Report” study (47) was used to frame the research questions. This survey was carried out in 2020 and administered to all registered coaches across Gaelic games, collecting data on demographics, coaching status, coaching experience, coaching practice, future coaching intentions, and coach education. In total, there were 11,569 valid responses, *n* = 2,189 were female, which were compiled into a report to inform this study. In sum, 68.5% (*n* = 1,490) of female coaches were aged 35–55, 86.3% (*n* = 1,885) were active coaches (i.e., coaching Gaelic games in the last 12 months), 32.4% (*n* = 607) coached across more than one code of Gaelic games, 2.6% (*n* = 48) were paid coaches and 7.5% (*n* = 139) were coach educators. Just under 20%, (*n* = 404) had no playing experience, 53.1% (*n* = 995) had less than 5 years coaching experience, 65% (*n* = 1,223) had none or foundation only level coaching qualifications, and 86.7% (*n* = 1,625) coached at grassroots in club settings. Major challenges for female coaches were balancing coaching with other demands (69.4%, *n* = 1,309), scheduling of competitions (31.7%, *n* = 597), increased bureaucracy (25.1%, *n* = 474), and lack of support from clubs/counties (18.7%, *n* = 352), while 52.4% (*n* = 962) were not engaging in further coach education due to lack of time. Despite this, 66.1% (*n* = 1,240) of female coaches would like to attain a higher coaching qualification.

### 2.2 Participants and settings

At the end of the survey used in the “Coaching and Coach Education in Gaelic Games: A Baseline Report” (47), respondents were asked to opt in for follow up to engage in focus groups. One hundred and thirty six, or 6.2% of the overall sample of, female coaches consented to this next phase of the research. Prospective participants were contacted by email to invite them to take part in the focus groups. Information sheets and consent forms were shared following expression of interest from participants with a cooling off period of 1 week before seeking confirmation of intention to participate. Of the 136 eligible participants, 27.9% (*n* = 38) subsequently participated in the focus groups. Participants were separated into the following categories for focus groups: Generic Coaches (4 focus groups, *n* = 19), who were actively coaching in Gaelic games in club/school/representative settings, Coach Developers (1 focus group, *n* = 5), who are coach education tutors, Full-time Paid Coaches (1 focus group, *n* = 4), who are employed by Gaelic games NGBs, Cross-Codes Coaches (1 focus group, *n* = 6), who coach across Gaelic football and hurling, or Inactive Coaches, (1 focus group, *n* = 4) who were previously involved in coaching Gaelic games but had not been actively engaged in the previous 12 months. This stratification of the sample gave greater coherence to the discussion within each group.

### 2.3 Data collection

Data collection took place in Summer 2021, where despite the ongoing Covid-19 pandemic, Gaelic games activity had returned to full capacity and was taking place in clubs and intercounty settings. As a result, prevalence of coaching and recruitment of coaches was not being impacted by Covid-19, which was not subsequently considered or integrated into the aims or objectives of this study. However, due to the pandemic and the all-Island demographic, it was not safe or practical to conduct the focus group interviews in person, therefore, all 8 focus groups were conducted using Microsoft Teams. Choosing to conduct focus group interviews gave an “open forum” for the participants to speak openly and honestly about their thoughts and opinions of coaching in Gaelic games. Dilshad and Latif [(53); pg. 191] stated that focus groups “yield shared understanding and several perspectives of a given topic” and they are known to be one of the most valuable tools for collecting qualitative data. The technique of using focus groups is a bridging strategy for scientific research and local knowledge (54, 55) therefore this method was selected as it offers a platform for differing paradigms or worldviews about a particular subject topic (56).

Focus group conversations ranged between 47 and 87 min in duration, with an average time of 65 min. The pre-determined questions asked during the interviews focused on variables considered, and findings from the “Coaching and Coach Education in Gaelic Games: A Baseline Report” (47) and on recent and relevant literature around females in coaching. The questions presented during the focus groups concentrated on several key areas of: (i) Coaching Enjoyment (ii) Perceived Challenges & Barriers (iii) Coaching Environment (iv) Coaching Confidence (v) NGB Support (vi) Future Coaching Intentions, and (vii) Reasons for Stopping Coaching. Each focus group was conducted by at least two members of the research team, with all members of the team supporting at least one of the 8 focus groups conducted. AG, TH and/or PD were involved in all of the focus groups for consistency. Team members who conducted the focus groups reviewed the recordings and transcripts to ensure responses were transcribed verbatim.

Prior to commencement of the focus groups a pilot focus group was conducted by AG, with support from TH and PD. Collectively, these three researchers developed and refined the topic guide, which was recorded and discussed before a final engagement with the wider research team to seek their feedback and approval to progress with the main data collection. During data collection, AG, TH and PD held regular meetings to discuss and reflect on the interview process and the data being accrued by the wider research team.

### 2.4 Ethical issues

Ethical approval from Ulster University's School of Sport ethics committee was sought and provided (2/6/21). This ensured the university guidelines and policies were upheld and maintained. The overall quantitative database is controlled by GAA personnel and is stored securely on their system. The custodians of the database gave permission to review and analyse the set of data to support this study. This phase of the study was prepared, designed, and implemented in collaboration with members of the GAA's research team, and all information obtained was safely stored by the host organisation according to GDPR regulations.

### 2.5 Analysis

Analysis was carried out using Braun and Clarke's (57) thematic analysis and was led by AG, TH, PH and PD with support at various stages from the wider research team. The first phase of analysis, conducted by AG, involved familiarisation and coding, which started naturally during the transcription phase. Coding was largely deductive or top down using the study objectives, and the EIM framework, as an overarching guide, similar to LaVoi and Dutove (9). Specifically, segments of text were coded sequentially in the transcripts by AG, with support from TH, PH and PD, with codes refined and reviewed as necessary to accurately reflect similar data. Semantic and latent coding were used to code using the language of the participants, for example around learning opportunities in coaching, but also to identify hidden meanings in text, particularly in relation to the cultural context of coaching. Adopting a primarily essentialist approach, codes were organised into themes and sub themes using the principle of recurrence and the EIM framework, subsequently arriving at 4 overall themes and 13 sub themes; Personal, Coaching Environment, Support and Learning and Societal that reflect the four layers of the EIM framework (individual, interpersonal, organisational, and societal). Again, these were developed by AG, TH, PH and PD before discussion with the wider research team. Lincoln and Guba's evaluation criteria (58) were used to establish trustworthiness in the data. Credibility, confirmability and dependability was assured through the engagement with the wider research team, which included presentation and discussion of the thematic analysis undertaken, with coaches and coach developers who have extensive experience working in female coaching contexts. In addition, the final thematic analysis was presented, and discussed, at a Gaelic games research seminar, which included coaches, leaders, players administrators. Given the very specific context of this study, transferability is challenging but the detail presented in the analysis, and subsequent assimilation with literature, will support other sports to consider implications, if any, for their respective female coaches.

## 3 Results

**Figure 1:**
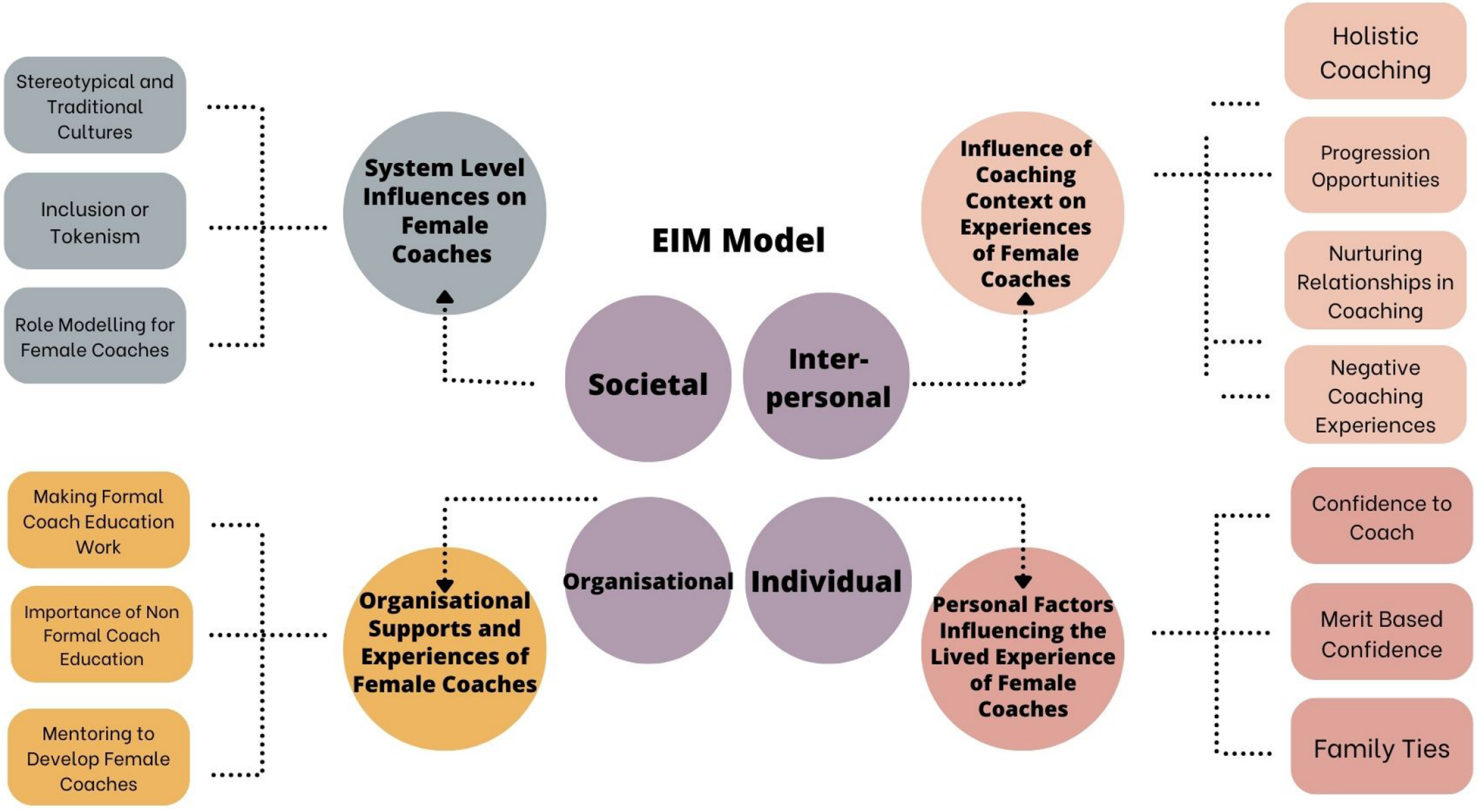
Overview of key themes and sub themes of lived experience of female coaches in Gaelic games.

Table 1 provides brief biographies of a sample of participants from each of the groups involved in the study. Pseudonyms are used here and across the results section. The 4 themes and 13 sub-themes are displayed in Figure 1. As noted earlier, the 4 key themes were methodically assigned, and examined in the context of, a specific element within the EIM framework: Individual, Interpersonal, Organisational, and Societal. The sequence of results reflect the authors decision to spotlight system and organisational level influences on female coaches before addressing individual level factors.

**Table 1:** Brief biographies of a sample of participants from each of the groups involved in the study.

Active coach	Mary Elizabeth's experience in Gaelic games began in primary school. She was from a small national school, so participated in Gaelic, hurling and camogie, with girls and boys. She indicated no boys played in the camogie team, but the girls had to play on the boys hurling and football teams to make up numbers. When she moved to secondary school she played table tennis, hockey and basketball. The reason she started coaching was because her son started to play. Team looking for volunteers and in her words “I think it is kind of boring just standing on the sidelines and it can be kind of cold as well standing there. You are supposed to be standing on the sides, so I said, there is no bother, I will jump in as well. But, I would have been conscious at the start that like my hurling and football, obviously very rusty, but you are kind of thinking how hard can it be with five year olds or six year olds, it can't be that hard. It is true, you learn along with them and it was fine”
	Sharon is still currently active in playing Camogie but actively coaches the U10 hurling team. Has been playing and coaching since she was young and then had a couple of children and took a bit of time out. Her eldest child is 13 and she is actively involved in club not just coaching but also administration roles within the club. In Sharon's word “So fairly full on”
	Katrina is a mother of four who now coaches the under 9s, her daughter's team. She has a background in and childcare and social work so has a lot of experience of working with children in groups and working with parents. She has indicated this is one of the reasons for her involved in coaching she has stated, “So that's kind of what I can bring to the table really”
Inactive	Lauren is not an active coach since 2019. She was coaching since she was 16–22, mainly with under 12 to under 16 groups, all in ladies football.
Cross codes	Deirdre has been involved in coaching for about 10 years, since her kids started mini leagues and she is now the juvenile coordinator for both football and camogie looking after all the teams from mini leagues up to minor, coordinating mentors and coaches and pitch allocations and trying to make sure in her words “we're not shafted by the men.” She has four children, three who play GAA (U16, U14 and U8) and another who plays Water Polo. Her husband is also involved in hurling.
	Fiona is a mum of three boys. They all play different sports. She has 3 children who all play sport under 13, under 11 and under 9 in GAA. I coach the under 11 boys and also coach my other son in soccer as well. Fiona also coaches handball. General background in sport is camogie and also football as a second sport. She stated it had been a long time since played sport herself, but wanted to get back involved and therefore got involved in coaching. She has coached senior teams in Dublin but has moved around.
	Maura has two boys and the club looking for people to help out with coaching, completing the foundation award and within a year completed level 2. She completed awards in two codes and stated she preferred hurling but coaches the two codes.
	Kerry spent all of her time when she was younger at the club as both her parents were involved. She did a foundation coaching award when she was young but it was when she completed her PGCE she began to coach. She has coached at a number of clubs both boys and girls.
Coach educator	Eileen is a coach developer for about 10 years with the Camogie Association. She took on this role as this was just something that interested her outside of her day job. When she applied to the Camogie Association she had completed a lot of different coaching courses for different sports as part of her college training and as part of her job and also her leisure time interest.
	Anne-Marie has been a coach developer for four years. The reason she wanted to become a coach developer was personal due to previous experience. In her words, “Not having the best coaches maybe just in the way that they were treating players and things like that I wanted to improve coaching.” So, she became a coach developer to encourage more women to get involved in coaching.
Full time	Louise has been in her current role as a Games Promotion Officer (GPO) for the last 5 years. She indicated she was late getting involved in GAA at 11 but stated she was lucky to have good coaching in school and a teacher who was very GAA orientated. She was 16 when she started coaching due to a bad injury. She indicated that her first coach delivered the foundation course she attained and from there she got involved in her club and has been coaching ever since.
	Aisling is a games promotion officer for the past 3 years. She was inspired to do this role due to the GPO who delivered in her primary school and got her involved in the sport.
	Jackie who has played football nearly all her life since probably under 8s. She started coaching when transitioning in secondary school.
	Aoife has played football from a very young age and as she sated this was the only option for her with regards opportunity for sport. It was Gaelic games or cross country running. She didn't start coaching until she came to the end of her own football career. She went to college and worked for 17 years as a medical scientist. In her late 20s, I was kind of getting a bit preoccupied about how I could get faster or keep myself faster when I was running. So, she ended up doing a fitness instructors course to support her understanding. Went on to complete a masters in Strength & Conditioning afterwards and had an opportunity to lecture part. Interest in youth athletic development led her to getting involved in tutor training in the LGFA and about 5 years ago gained a role as a GPO.

### 3.1 System level influences on female coaches in Gaelic games

This theme is linked to the societal level of the EIM framework and is associated with the cultural norms that guide people's expectations, perceptions, and behaviours within the social groups of Gaelic games. This theme identified many considerations regarding the cultural context of Gaelic games and how this relates to coaching and had 3 sub-themes which are detailed below with additional context in Table 2.

**Table 2:** System level influences on female coaches in Gaelic games.

Stereotypical and traditional cultures within the Gaelic games	“There was a bit of a perception for a while that, ‘Oh well, we're only a Camogie club and you have to be off the pitch by…’, and I said, ‘By the way, that 500 Euro cheque, you're not getting it this year!’ So, no-one spoke, and we soon got our allocated spot.” (P3, Active FG3)
	“It's a prevalent issue that's been there over the years in terms of lesser recognition for ladies’ football versus the men's football, and clubs. And so, it's creating that culture of acceptance and inclusion. There's a stereotype.” (P2, Inactive FG)
	“I do find it's very male orientated, the culture of men, they were running the show.” (P1, Inactive FG)
	“I think it's just that a lot of the men want to just keep it the way it is. Because it suits them. You know, it's just a historical thing.” P4, Inactive FG)
	“In relation to the chairperson of the club, we've never had a female, they've always been males, but the females get to do the Secretarial role or the Registrar role.” (P2, Active FG3)
	“I think females can offer a few different things in terms of empathy and maybe understanding the social situations and different things like that.” (P2, Full Time FG)
	“I notice that the male coaches are more competitive than I am, we have our own little mini All-Ireland tournaments that plays every summer and all the coaches, we mentor different teams, they're all playing against each other, and I'd be much more about making sure everyone's getting a touch.” (P1, Generic FG3)
	“The men do the coaching, and the women make the tea and sandwiches, and clean the jerseys and the bibs.” (P1, Active FG1)
Inclusion or tokenism	“It is really just about the respect, about the men listening to your voice. Your voice is just as good as theirs and you're doing the very same job as they’re doing.” (P5, Active FG2)
	“I was at the Coaching Conference for the last couple of years and females are very much the minority out of major, major numbers of our male counterparts at the GAA conferences.” (P3, Inactive FG)
	“I think around a group of men sometimes, the female voice is the one lone female voice. You don't like to be taken as a token chaser, as a female, we can stand up.” (P4, Active FG2)
	“The men do the coaching, and the women make the tea and sandwiches, and clean the jerseys and the bibs.” (P1, Active FG1)
	“We've a very good environment between the male codes and the female codes. I'm heavily involved with it at committee level as well. So, the environment I'm coming from is very good. It's very supportive of the female coaches.” (P4, Active FG2)
	“Our club has very much been pushing for more female involvement, both on players and coaching staff. And you can see right the way up that there would be females coaching practically all the teams.” (P1, Active FG3)
	“Especially from being involved in LGFA side, you know, it's very male dominated and you would expect it to be more female dominated and I set up the nursery. I brought parents in to help out but I've never played Gaelic in my life, I'm not a Gaelic person, I'm a Soccer person but the Gaelic Association is just around the corner from me and that's why I got involved.” (P1, active—FG1)
	“With sometimes as a female, your voices aren't always heard.” (P3, Inactive FG)
Role modelling for female coaches	“You need role models in place in clubs and a County to get that younger generation in so that it starts from that age rather than when you’re older.” (P4, Inactive FG)
	“I love the GAA, it's a passion, it's a motivation. I'm very competitive. I went into coaching, didn't realise it was my forte and I had really good role models who coached me, and giving back to the club.” (P3, Active FG1)
	“Our club has done a big drive over the last two years to just increase the female numbers and we had a target set for the 2020 year, ‘If she can't see it, she can't be it’. And we grew by the female membership grew by over 300%.” (P5, Active FG3)
	“The biggest barrier, is trying to get through to the male coaches that females and males do not react the same way to a win, they want the participation, they want the enjoyment, and that, we, as coaches, are there to facilitate the players.” (P1, active—FG1)
	“Even having more females involved with inter-county teams as well going forward, I think that would be something to really inspire the future generation of coaches and maybe children that we are coaching right now, that they can actually take the coaching and if they have had a positive experience of GAA, hopefully they can than take that with them and inspire the next generation.” (P1, Full Time FG)

*Stereotypical and traditional cultures* related to the structures, hierarchy, and power within Gaelic games, across NGB and club settings. Many female coaches remarked on a male-orientated culture or an “old boys club” within the organisational structures of Gaelic games.

“I do see those barriers in relation to the, I suppose, the authorisation and the autonomy within the club. I think that the hierarchy there is very much male orientated. I mean our constitution still only refers to ‘he’. So, you know, things like that, it's as if the word ‘female’ or ‘woman’ hasn't come into effect.” (Katrina, mother of four who coaches an U9 team)

“Because I've been in the position for a long time, I've actually been dealing with people, the same people fundamentally, for a very long time and I've always, like there's people you get on with and I've never had a major problem with—I've had one or two lads now who are a little bit aggressive but most of them are fine. But I will say that within the organisation, I think even within your own club, there are men who bring an aggression I think, is probably the word and a lot of baggage. And they've a long, long way to go in terms of how they deal with women. And that is ingrained, that is just part of culture and that is difficult and not everyone is going to accept that.” (Eileen, coach developer)

This culture was also evident in coaching contexts, with female coaches observing how male coaches have a different style and philosophy in their coaching.

“A barrier that I've had is I coach with six other coaches, I am the only female, the rest are all male and I have found through coaching with different coaches along the way up, where I've always been the only female coach, that one of the biggest things is for me, anyway, from my experience, is that the male coaches tend to look at the girls and want to relive their experiences through the girls and not necessarily look at the girls and see how the girls can develop as individuals.” (Lauren, mother of three)

There is a stereotypical perception within Gaelic games that females are less knowledgeable and less competent within a coaching context. This was a major challenge noted by the female coaches as they felt the need to constantly prove themselves in a coaching context. They were often allocated “feminine” or caring roles within the coaching set-up, while males were presented as the manager, coach or the “person in charge”.

“The attitude of males towards female coaches and some of that might be unconscious bias that they don't realise that they treat female coaches that way and other times it might be out of sight out of mind that they don't think of females in their sport.” (Louise who has been a GPO for the last five years)

This culture has often left female coaches struggling for equal access to facilities within their clubs. It was not just Paula, an active coach of 4 years, but other coaches within her focus group who agreed with her comment; “So, we have to discuss, and sit down with the men's club, as to when we are allowed a pitch and all of that. So, you kind of get pushed down.”

This persistence with ‘the way of doing things” is a barrier to the recruitment and progression, as well as a challenge for existing female coaches in Gaelic games.

*Inclusion or tokenism* referred to female coaches feeling valued and respected in their coaching roles. Many female coaches highlighted a lack of respect, credibility, not being taken seriously as a female and feeling undervalued, and underrepresented. These challenges ranged from their voice not being appreciated as strongly as a man's voice when coaching, to not being included in team specific or broader club coaching activities.

“It's really the ‘old boys club’—they share information with each other, but don't share with everybody. Information is not shared equally between members of the club or coaches.” (Aoife actively coached for six years until Covid)

“Very often you were the ‘token female’ there, you were brought in because they needed a female rather than actually valuing your input.” (Ashton, active coach who is still playing ladies gaelic football)

There were some positive instances of clubs working to be inclusive in their coaching model. Sarah one of the active coaches discussed her club stating, “Our club has very much been pushing for more female involvement, both on players and coaching staff. And you can see right the way up that there would be females coaching practically all the teams.”

Finally, *role modelling for female coaches* was endorsed by participants who emphasised the need to act as, and avail of coach role models within Gaelic games to inspire young female coaches.

Colette, a coach developer for over ten years stated, “I've three male mentors working in with me and they're reasonably supportive but I mean, again, they're volunteers, they're not there all day. But you have to deliver as a leader, you have to kind of defend the position of the team. You've got to make sure that we get access to training slots, that we have access to our players for matches which is not always a given. And I have found that very difficult to deal with but I think within me, I am probably at this stage, there's day that are harder than others but I’m a tough old bird and I have the support to discuss it.”

“I would like to see a lot more of the female managers, the female coaches. I'd like to see them on TV, I'd like to see them being interviewed and I'd like to them at the coaching courses, leading out some of the foundation courses, the coaching courses in every County.” (Sharon, an active coach and involved in club administration)

This was endorsed by Noleen, a coach developer who commented that “once you know the content it probably is more in my favour being a female delivering on the camogie coaching courses.”

Coaching remains quite gendered, and it was evident from the data that role models could be used to work against this and recruit more females into coaching.

### 3.2 The role of organisational supports in the experiences of female coaches in Gaelic games

This theme was linked to the organisational level of the EIM and relates to the NGB and club-based context for coaching within Gaelic games. This theme had 3 sub-themes, described below with additional detail in see Table 3.

**Table 3:** Organisational supports to improve the experiences of female coaches in Gaelic games.

Making formal coach education work	“And they're all volunteering three days a week, I think there has to be some way of the CPD thing that we're trying to do, or you know, the in-service, that maybe mandatory things like that, that you have to attend, or you have to build up so many credits’’ (P2, Coach Developer FG)
	“I think some of them should nearly be compulsory for all coaches because they're not teaching you how to teach but they're teaching you little tricks and tips’’ (P1, Active FG2)
	“We're time poor, please, don't be expecting us to be on every week, for two hours, this has to be coordinated—so they have to be appropriate, timely, and have to be effective and efficient.” (P5, Inactive FG)
	“For Cul Camps, we were given a booklet of drills that would be good for the camp. But I did the 2 coaching courses, and I didn't receive any of those kinds of resources. I think a lot more needs to be done on coaching pedagogy and developing pedagogy aspect of coaching.” (P1, Inactive FG)
	“For foundation courses, they did a series of Go Game workshops. And they sent on the slides and handouts and drills. So, I think access to those resources is invaluable and also tie it up with the practical work.” (P2, Inactive FG)
Importance of non-formal coach education	“Online settings, especially from a female, or being a mother as well, and a family background. The courses, in the past, have been quite centralised. A sort of a general forum like this just for various things to get hints of things, or what issues that other people are finding, I think would be great, without sort of a formal setting.” (P4, Active FG1)
	“Once a month we used to go down to a pitch on a night and they might get, it might be just our GPO or it might be a visiting GPO or like a guest star coming down to do a coaching course and so on like that, just a one night thing, something practical. Or if somebody knows—I remember one time they got Sinead Finnegan down and she did a session.” (P3, Active FG2)
	“Coaches Forum and it's open to LGFA and GAA. And during lockdown, every two weeks, we did a Zoom session where there'd be a video shown, we'd all discuss it or something like that.” (P3, Active FG1)
	“And all these other programmes now, due to Covid, this is fantastic, Micro soft Teams because we as coaches, wouldn't have time to do this here. Sharing your knowledge, your experience through forums, through networking and then also having those networking days specifically for females.” (P1, Active FG2)
Mentoring to develop female coaches	“Shadowing and mentoring. And even that learning of shadowing to buddy up, I learnt an awful lot. I asked could I turn up to their training sessions.” (P3, Active FG2)
	“My husband is a professional coach in rugby, but I would ask him various elements as well, because he would have read up on all the different areas. I would actually consult with him on different things.” (P1, Active FG3)
	“I think experienced coaches should be mixed in with the inexperienced coaches and we're very lucky obviously in my coaching in that we have that mix.” (P4, Active FG3)

*Making formal coach education work* relates to the various levels of coach education courses, support and learning offered by NGBs via their online platforms. CPD courses and qualifications within Gaelic games are not a compulsory requirement for coaching, consequently coaches can coach with little to no training at all. Several females stated the need for specific compulsory workshops to be implemented by the NGBs.

“I suppose you can't make (all) the courses compulsory because people are volunteering their time but, I mean we all have to do our fundamentals, we all have to do our safeguarding. There should be specific courses or workshops that coaches have to attend.” (Mary Elizabeth, who played hurling, football and camogie in her younger days)

Formal education, that is planned, designed, and organised with a certification process, is valued, but also a challenge for female coaches due to the time commitment involved.

“I think we need to look at the way the coaching courses are set out, and the way they're delivered, and the timing of them, to make them more family friendly for mothers. Probably one of the best things I think from lockdown has been the online courses that can be done, and I think they should be pushed and encouraged, because it probably does help from a female point of view.” (Collette, who due to family commitments is no longer actively coaching)

The female coaches also stated the need for “Sport Specific Courses” focusing on areas like technique, skills, tactics and teamplay as well as the development of a “learning culture” and for additional resource information (e.g., booklets, hand-out sheets etc.) to be made available post-courses.

This learning culture was also reflected in the sub theme “*importance of non-formal coach education*”. Within Gaelic games non-formal opportunities arise in the form of online courses, forums or webinars. Female coaches highlighted the importance of this online “community of practice” to share knowledge and ideas, particularly female only networks. Several coaches favoured online coach education in general, seeing it as a constructive and highly beneficial development for female coaches.

Having things like this. This has been great. Me speaking to other people from other clubs. I'm after getting loads of ideas today, and loads of views, and some people are exactly the same as us, or, that club is different than us. It's not just hearing something on the pitch, or hearing something here, and there. This is actually great.” (Katrina, mother of four who is actively coaching her daughter's team)

Full time coaches were very supportive of community of practice models in club settings. Jackie said that: “I think club coaches should meet up maybe twice a year, sit down in a room or stand at the corner of a pitch and do exactly what we did during lock down there and bounce ideas off each other. What worked, what didn't work, what went well. What would we do to improve our sessions and I think trying to keep everything kind of positive because sometimes I find when you bring all the coaches together it can be a case of ‘I did this and this didn't work’ or ‘I done the other and it didn't work’ and it becomes a kind of everything is going wrong kind of thing? So, if you bring it into a positive environment, tell me what's going well and get the feedback off the coaches and bounce ideas off each other and it will build a bit of rapport within the club as well.”

Within the informal learning space “working with or observing other coaches” and “reflecting on own and others coaching practice” were deemed important coach learning tools. *Mentoring to develop female coaches* reflects how female coaches valued mentorship, peer coaching or co-coaching within Gaelic games as supports for continuous learning and development.

“I think experienced coaches should be mixed in with the inexperienced coaches and we're very lucky obviously in my coaching in that we have that mix.” (Fiona, who is involved in coaching across various codes)

Female coaches also enjoyed forming friendships and professional relationships with other coaches that they felt comfortable sharing ideas with and soliciting feedback from.

### 3.3 The influence of the coaching context on experiences of female coaches in Gaelic games

This theme relates to the interpersonal layer of the EIM, and the connections and relationships between people in coaching, which in this instance includes peer coaches, parents, and players. This theme had 4 sub-themes, presented here and in Table 4.

**Table 4:** The influence of the coaching context on experiences of females coaches in Gaelic games.

Holistic coaching	“For me, it's not only the development of their skills on the pitch, but also really important to see the development of the individual and how Gaelic Games has actually assisted them in developing their own personality and becoming more confident in themselves.” (P2, Generic FG3)
	“A successful coach would make a session fun, they learn their skills, they want to come back and it gives them confidence I suppose. I heard a quotation, ‘You don't remember what a coach said, you remember how they made you feel.’” (P1, Generic FG2)
	“For me a successful coach would be like what Jackie said, that your numbers are good at training all the time, you're playing want to come back, they look forward to your session, they come to your session with a smile on their face even if they are teenagers. Yeh so that you're not actually driving people from the game and they keep coming back and that they are learning something in every single session, even a small thing but that to me would be a successful session and then that you just see that gradual improvement over time and it does take time.” (P4, Full Time FG)
Progression opportunities	“I'd like to progress with the girls, because I'm amazed at how much they have come on in their short years already. They absolutely amaze me at how quick they learn things, and how confident they become, it's brilliant.” (P3, Generic FG1)
	“I want to manage a senior men's team, hopefully there's a club out there somewhere that will believe in a woman's coach, at least that we can do the same as any man, that we inspire them the same, that we can show them the experience we have.” (P1, Generic FG1)
	“If you went seriously into our senior hurlers or senior footballers, and said, I'd like I think you'd be laughed out of the gates unless you were coming in with a half dozen All-Ireland medals in your back pocket from camogie or football then it would be jog on” (P1, Active FG3)
Value of relationships in coaching	“We've a very good relationships between the male codes and the female codes. I'm heavily involved with it at committee level as well. So, the environment I'm coming from is very good. It's very supportive of the female coaches.” (P4, Active FG1)
	“I just think language is so important in terms of coaching and it's probably where everyone should start because, your voice is the tool you're going to use the most. It's the language that coaches use, that their message is clear and concise.” (P2, Active FG2)
	“Communication's a massive one. Because if you're able to communicate properly to them, you will be more effective in a polite and obviously respectful manner.” (P1, Active FG2)
Negative coaching experiences	“I have an issue within a club level where I have a full female coaching team, and now I've had a male parent while he was walking past them, refer to them as ‘stupid bitches’—and they would never have said it if it was a man. So, I think even some men and some parents think it's okay for them to demean a woman at some times.” (P2, Active FG2)
	“I won't lie to you, there were days where you feel like it's all obligation and no rights, and that's very difficult to deal with, I mean really difficult to deal with.” (P3, Coach Developer FG)

*Holistic coaching* referred to the importance of taking a player-centred and whole person approach to coaching and supporting player autonomy in sport. Female coaches in this study attempted to apply a player-centred approach, through prioritising fun and engagement.

“You have to look for your definition of success and I'm maybe speaking a bit here but success, what is success? Like, you might possibly, if you only coach one team, get one person, ever, to an Intercounty. But that's not a given, is that success? If that's success, you may never see it. Or is success getting 20 or 40 girls staying playing football until they're 16? I think that's success. Getting them to understand, getting them confident, showing them that they can play football, it's not just for boys. That, you know, there's a community, there is support networks for them themselves to build. So, it's back to this, you know, girls building networks and having a strength of a network there to support themselves because sometimes they're going to need that.” (Anne-Marie, coach developer)

Ashton, an active coach, observed that “I've seen girls’ confidence grow from not being able to say ‘Hello’ to you on the pitch, to all of the sudden now giving me cheek and I think that's great because that means they've grown as individuals which can only make them better players because they'll be stronger on the pitch.”

It was noted earlier by participants that male coaches often have a different coaching style that emphasises winning and competition.

*Progression opportunities* related to the opportunities available for female coaches to progress within their Gaelic games coaching career. While findings showed that female coaches tend to progress in coaching as their children advance in sports, other female coaches expressed an ambition to coach male teams in the future but felt that opportunities were not available to do so.

“I intend to go to the senior level within my club, I am just going to go with my children and if they drop out, I'm still going to go.” (Katrina, mother of four who is actively coaching her daughter's team)

“I think there needs to be a better structure, to identify really good coaches and to ensure they're pushed on along up to, but if someone does show promise or show ambition, or is good in certain areas they need to be identified.” (Imelda, recently involved in coaching with a teaching background)

*The value of relationships in coaching* indicated how females felt it was important to have strong, trusting relationships between coaches to ensure collaboration, cooperation and teamwork. Female coaches remarked that relationships are influenced by good communication and challenges arose due to breakdown in communication. This caused problems between individuals leading to hostile coaching environments that ultimately impact engagement and retention of female coaches.

“I just think it's about feeling like you have an equal voice and an equal say and just having that dialogue with people who are your equal and ensuring that you feel valued.” (Siobhan, whose husband is also involved in coaching)

Many of the female coaches noted positive experiences in fun and enjoyable coaching environments. However, several coaches highlighted *negative coaching experiences* in the form of abusive or derogatory comments made by male coaches or parents.

“I had poor coaching experiences as a player and a coach as I was getting abusive phone calls being like, ‘Why aren't you making training?’ I had to notify them a week early. I was like, nah, I'm not going to continue. It was also kind of parents as well.” (Natalie, who was involved in coaching but is no longer active)

### 3.4 Personal factors influencing the lived experiences of female coaches in Gaelic games

This theme, which reflects the individual layer of the EIM is associated with female coaches looking at the individual person or self to describe their coaching experiences. Key data from the coaches are presented here and in Table 5.

**Table 5:** Personal factors influencing the lived experience of female coaches in Gaelic games.

Confidence to coach	“We do need to fix the women, we talked about developing their confidence, self-esteem and sense of belonging of being female coaches and being respected in a club.” (P 2, Inactive FG)
	“As a woman, I know we don't sell ourselves, and we don't rate ourselves as much as the male coaches within our sport.” (P2, Active FG1)
	“You're always kind of second guessing yourself. I would always be having that inner dialogue with myself around my ability as a coach—I would say, ‘Well, here's my evidence to prove to myself that I do have these skills, I've done this in the past.’ But as a female you do question yourself.” (P1, Active FG2)
	“A kind of imposter syndrome—If you don't feel competent, you need to act it, and then it comes with the whole, ‘oh look, okay, I can do this, I’m well able to do this’”. (P1, Active FG1)
Merit based confidence	“Extra confidence comes from knowing what the rules are in the game. It's knowledge of the game and you can get a bit more respect for it as well.” (P4, Inactive FG)
	“I felt like that was kind of what was my role, was coaching. So, I went and done that course and I got loads of experience from the college….from actually hands on coaching and even just the knowledge of different coaches coming in and even classmates and stuff like that. So, it was just excellent in that sense.” (P2, Full Time FG)
Family ties	“But I suppose being a parent you are kind of doing it for your own kids. Then you see what happens being a coach and you see when other kids start progressing.” (P 2, Inactive FG)
	“We are all doing it because we love to see our kids to well. We give them all individual attention and develop them, and they have their fun and their friends, and they develop their self-esteem and resilience.” (P1, Active FG4)
	“I've a family myself, and it is hard work getting out onto the pitch and getting everything juggled between home life and camogie coaching life.” (P4, Active FG1)
	“I suppose I just started not having a clue about coaching and it's the usual you go up with, I've 2 boys. They were looking for people to help out with the team” (P3, Active FG1)

*Confidence to coach* was a significant factor in the development of self-worth for female coaches. Responses indicated that, overall, female coaches had medium to good levels of confidence in their own coaching practice.

Paula an active coach who has been involved in coaching football since her children were introduced into the sport said, “Confidence is a big thing within a coach. Females just don't think they have the skillset or the knowledge to coach. It helps the confidence of the female coaches to know that you do know what you're doing, and you are every bit as good as your male counterparts.”

However, it was noted that lower levels of confidence were evident when dealing with male coaches or male parents. This suggests that male-dominated coaching environments created self-doubt and insecurity, and that some females struggle with confidence in the context of masculine hegemony. Lauren one of the coach developers stated, “there's days it's very hard to deal with and I think if you are not terribly confident, I think it could be quite damaging. You have to fight your corner. There will be people who’ll see you as a soft touch and try and manipulate you and make you move. And I've been in some pretty aggressive situations which, you know, you have to remember here that we're all volunteers.”

In contrast, confidence was significantly enhanced through positive reinforcement, recognition, and praise, particularly from parents and other coaches, as well as seeing players develop and improve through their coaching. This *merit-based confidence* developed from previous playing, and academic experience and ultimately accrual of coaching experience.

“Being a player. That helps me, so, I think experience is a great thing.” (Jackie, full time coach)

“I have a reputation of being a Sports Scientist, of having the qualifications, of having the background of working in those High-Performance areas. So, I was given the respect that most other people would be expecting—male or female, you had that reputation. That built my confidence and belief in myself.” (Aoife, full time coach)

Laura, a full-time coach observed that “It's a combination of education and it's a combination of experience. So, I think both of those things, and when you're doing it so often. So, like, you've built up a huge bank of experience very quickly. So, like, I would coach maybe 30 h a week, whereas your regular volunteer coach will have, maybe their team maybe two or three times a week, so they're getting maybe 3 or 4 h, maybe whereas I'll do 25 h in the schools, and then I'll have another, you know, to 2 to 3 h with the under 16 s.”

These experiences helped females to feel more respected and validated as coaches amongst their peers.

*Family ties* was an important sub-theme in this assessment of personal factors that impact female coaches. Family was perceived as, both a barrier and as a “gatekeeper’ to coaching. Female coaches indicated the difficultly of juggling family life, household chores and work commitments with volunteer coaching. At the same time, having children was a strong access point for females into coaching.

“As a mother, I think that is also a challenge. And I'm a working mother and I, I'm a principal of a school and all my kids are young. So, time is a big challenge for me as well and giving it enough, and always having that feeling of ‘God, am I given this enough?’” (Sharon, active coach)

“It is just commitment. I remember somebody saying to me once that girls will organise their lives around training and boys will organise training around their lives. It was that thing of the priorities.” (Katrina, active coach)

It was notable that female coaches often drifted away from sport if their child opted out of participating, suggesting familial connections alone were not sufficient to retain females in coaching.

## 4 Discussion

The primary aim of the current research was to investigate the experiences of female coaches within Gaelic games. Findings highlighted many barriers and challenges for female coaches that are personal, interpersonal, organisational and societal in nature. This section will now reflect on these findings within the broader research context to address the study objectives, as well as exploring limitations of the research, possible opportunities for further research and implications for policy and practice.

The overarching finding in this study is the influence of the socio-cultural context of Gaelic games on female coaches, and how this influence pervades across all layers of the EIM. For example, Jowett (59) emphasised personal relationships (e.g., coach–parent, athlete–athlete, coach–coach), as the foundation of coaching, and in this analysis, female coaches spoke about relationships in terms of dealing with aggressive coaches, challenging parents, and sexist behaviour, which is consistent with previous research in Ireland (39, 60). There is also clear resonance to work by Norman et al. (18) who highlighted the prevalence of sexist behaviour by male coaches towards their female counterparts. These interpersonal experiences of female coaches are not surprising when considered within participants discussion of coaching in a masculine oriented, traditional, “old boys club” environment in Gaelic games, presented separately as societal level influences in ecological models. Overall, such behaviours and experiences remain deeply rooted in the social norms of society and results in female coaches having a sense of being second best in sporting contexts (33, 61). Similarly in this study, female coaches felt undervalued, unappreciated, and self-referred as a “token female”, which Norman (62) observed leads to female coaches feeing they must work harder to prove their coaching competence.

Children and family appear to be the key access point for females to start coaching in Gaelic games and data showed that females usually remain in coaching while their children are involved. Participants mentioned the enjoyment and satisfaction of witnessing their children, and other kids, playing, having fun, engaging with others, and growing, and developing holistically. Previous research has similarly shown how parent coaches value the quality time their role gives them with their children, and the opportunity they have to develop sport specific and life skills in youth (63, 64). However, female coaches often have to “juggle” these mother and coaching roles (64) expressed in this study as managing “real-life issues” of household duties and domestic caring responsibilities while also finding time to engage in and prove themselves in coaching. Time is a challenge for all coaches in Gaelic games (47) and other sports (39), but is exacerbated for females who are faced with balancing family life, and the additional load they carry therein, and work commitments with volunteer coaching (9). Females also indicated their difficulty in attending coaching courses and programmes due to the associated time demands, which can hinder their learning and development, and opportunities to build confidence in their coaching (65). In turn, it is perhaps unsurprising that female coaches in this study, and elsewhere (9) have low perceived self-efficacy, skills, knowledge and assertiveness in their coaching.

Interestingly, however, female coaches in the study remarked on their holistic approach to coaching that often conflicts with the outcome-oriented styles of their male counterparts (66). However, this preferred coaching style of females is reflective of autonomy supportive coaching, which has been shown to enhance performance, participation and motivation in sport (67, 68), and indeed is the core of player and coach development in Gaelic games. Despite this, as in other research, the male voice and approach often prevails (33). In general, there remain clear challenges in coaching around unequal and misguided expectations relating to competence (35), which perhaps renders the lack of self-confidence and self-efficacy observed in this analysis as equally misguided, fitting a stereotype of what is expected in sport (69). At the same time, female coaches in this study are very merit oriented in their self-assessment of their coaching competencies and are ambitious in their coaching. This is a juxtaposition of sorts as meritocracy is not always valued in sport (70) and progression opportunities for females in coaching are often limited (18, 42), largely due to the default “think coach, think male” narrative in sport (71). Finally, coaching practices within Gaelic games remain somewhat stereotypical and traditional with findings in this analysis showing that females are still occupying gendered roles in coaching, such as taking on caring and pastoral responsibilities. Hogan et al., (48) observed that there is still some distance to go before female coaches in Gaelic games are universally accepted as a cultural norm and not impacted by an inherent bias, even if unconsciously on some fronts.

A core focus of this work was to use the female coach voice to inform supportive actions to help them thrive in sport (20). In turn, there are many lessons for Gaelic games policy and practice from this research. Firstly, the majority of female coaches in Gaelic games are coaching at grassroots and have introductory level qualifications. This research has confirmed that many female coaches have ambitions to progress in their coaching therefore understanding a coach's attitudes, subjective norms, and perceived behavioural control will support and predict intentions of those involved in coaching to progress along the coaching pathway. Therefore, as in other sports in Ireland (40) and elsewhere (72), Gaelic games must work to increase the participation of female coaches at all stages of the player pathway. To this end, in 2023, the Gaelic games NGBs launched a female coach mentor programme, which has extended in 2024 to a female sports science practitioner mentor programme. The focus of these initiatives is to increase the representation of females in coaching at talent and elite stages of Gaelic games (73). Mentorship programmes have many benefits as they focus on improving knowledge, skills, confidence, and experience, individually, and developing a support system, interpersonally (9). Recently, Taylor and colleagues (72) presented organisational practices that could attract, develop and retain high performance female coaches. These included networking/mentoring but also emphasised the need for flexibility in coaching opportunities, building career pathways, role modelling and delivering bespoke education for female coaches. Indeed, Banwell et al., (32) in a review of a female coach mentor programme noted that despite positive experiences, participants felt little or no impact on the wider organisational culture of the sporting organisation and how it supports women in coaching. It is important that Gaelic games consider the breadth and depth of their efforts to increase the representation of female coaches across the player pathway.

Secondly, this study has confirmed that women are often marginalised in club contexts and conversely that positive coaching interactions and good relationships with other coaches, parents, and players helps coaches to stay connected in coaching with subsequent feelings of support and opportunities to learn from others supporting women to be involved in coaching (74). Previous research has shown that female coaches in Gaelic games have emphasised the need for initiatives to support the recruitment, development, and retention of female coaches in club settings (48). There are several considerations from this study that could inform decision making and practice in Gaelic games in this area.

(i) Female Coach Network: In developing the full suite of coach learning opportunities, it is recommended that a targeted female coaching network is created and implemented. This will encourage, direct, and guide female coaches to participate in coach development programmes. In addition, such a network will assist female coaches to progress through the appropriate coaching pathways, increasing their competency, self-belief, and credibility. This recommendation aligns with previous studies that reported how women preferred single gender courses as they often felt inferior or uncomfortable among male attendees (75, 76).
(ii) Holistic coaching environments: there appears to be systemic level issues in Gaelic games, and elsewhere (6, 77) that sustain stereotyping, gendered roles and, in some instances, sexism at national and local levels. Sport leadership must set standards around coaching to promote inclusive, holistic, supportive coaching environments (78).
(iii) Establishing structured programmes to support clubs in identifying, selecting, and developing strong female coaches. By supporting the recruitment, development, and retention of female coaches the Gaelic games Associations will challenge the stereotyping and gendered roles traditionally associated with female coaches. Thus, leadership within the Association will challenge their units to set standards around coaching to promote inclusive, holistic, supportive coaching environments (78).
(iv) Flexible/family friendly coach learning: female coaches have many challenges around time, which hinders their ability to engage in coaching and coach education (9). Coach learning opportunities should be offered online, so that it is easily accessible and/or in family friendly contexts with childcare available (79).
(v) Communities of practice: female coaches favour peer learning and the development of support networks, which could be facilitated through “communities of practice” (80, 81). These forums support female coaches to generate ideas by discussing, engaging, and learning from each other, in turn increasing self-assurance and self-confidence levels within their coaching abilities (82).

### 4.1 Limitations

While this research provides an insight into the experiences of female coaches within Gaelic games, it is not without limitations. Firstly, as the data are self-report and generated from a convenience sample of participants who self-selected to take part in the focus groups, therefore the experiences of the participants should not necessarily be viewed as representative of all coaches in Gaelic games. Analysis was largely deductive, albeit using a commonly used framework in similar coaching research. The authors recognise that other frameworks may be used as a lens to understand the experience of female coaches. Finally, the Gaelic games setting is quite unique, which may impact the generalisability of findings to other contexts or codes. While previous coaching research has appreciated the importance of context, it has tended to treat context merely as a resource for analysis, rather than influencing situated practices and formal coach education (83, 84). It is hoped that this research may resonate with coaches and researchers in other contexts and be replicated across other sport settings, while also being impactful within a Gaelic games setting.

## 5 Conclusion

This research provides an in-depth investigation into the lived experiences of female coaches across Gaelic games. Overall, the findings support previous investigations that have highlighted the lack of female representation in sports coaching and the many multifaceted barriers that prevail for females in coaching (12). Female coaches in Gaelic games experience many of the common, albeit nuanced by gender, challenges for volunteers in sport, especially around time; both to actively coach and to engage in coach learning. They also experience many organisational and cultural barriers that are systemic in nature, such as poor flexibility in coaching/coach learning, lack of respect, stereotyping, and limited progression opportunities. Collectively, this creates a gendered environment for females in coaching in Gaelic games with a cyclical impact of organisational issues that ultimately effect the representation, confidence, competence, and progression of females in coaching in Gaelic games.

It is important to note that the intention of the Gaelic games associations is to promote integration and inclusion through increasing awareness of unconscious biases which may be removed or at least reduced (48). This indicates that the principles underpinning coaching and coach development in Gaelic games are solid but, as in other sporting contexts, they need to prioritise accountability across all units (20). At the same time, the coaches voice in this analysis is consistent and coherent, and calls for the development of a bespoke, system wide strategy for female coaches in Gaelic games or as Norman and Simpson (85) has stated it is time to move to a stance of action.

## Data Availability

The raw data supporting the conclusions of this article will be made available by the authors, without undue reservation.
